# 1876. Characterization of patients with active drug-sensitive tuberculosis on synchronous and asynchronous video monitoring treatment in Cali - Colombia during 2019-2022

**DOI:** 10.1093/ofid/ofad500.1704

**Published:** 2023-11-27

**Authors:** José Fernando García-Goez, Sofía Alexandra Montes-Tello, David A Rios-Pineda, Esteban Uribe-Romero, Mayra A Rojas-Ramirez

**Affiliations:** Fundación Valle del Lili, Cali, Valle del Cauca, Colombia; Fundación Valle del Lili, Cali, Valle del Cauca, Colombia; Universidad Icesi, Cali, Valle del Cauca, Colombia; Universidad Icesi, Cali, Valle del Cauca, Colombia; Universidad Icesi, Cali, Valle del Cauca, Colombia

## Abstract

**Background:**

Tuberculosis (TB) is an infectious disease with a high burden of morbidity and mortality, potentially curable in up to 85% of cases with first-line treatment, when adherence is over 90%. Direct observed treatment (DOTS) guarantees treatment adherence. However, barriers such as the time and cost of travel to the supply site, social stigma have weakened its efficiency. As an alternative, video monitoring of tuberculosis treatment (VDOT), had been evaluated with favorable results compared to DOTS, making it a promising strategy.

**Table 1.**
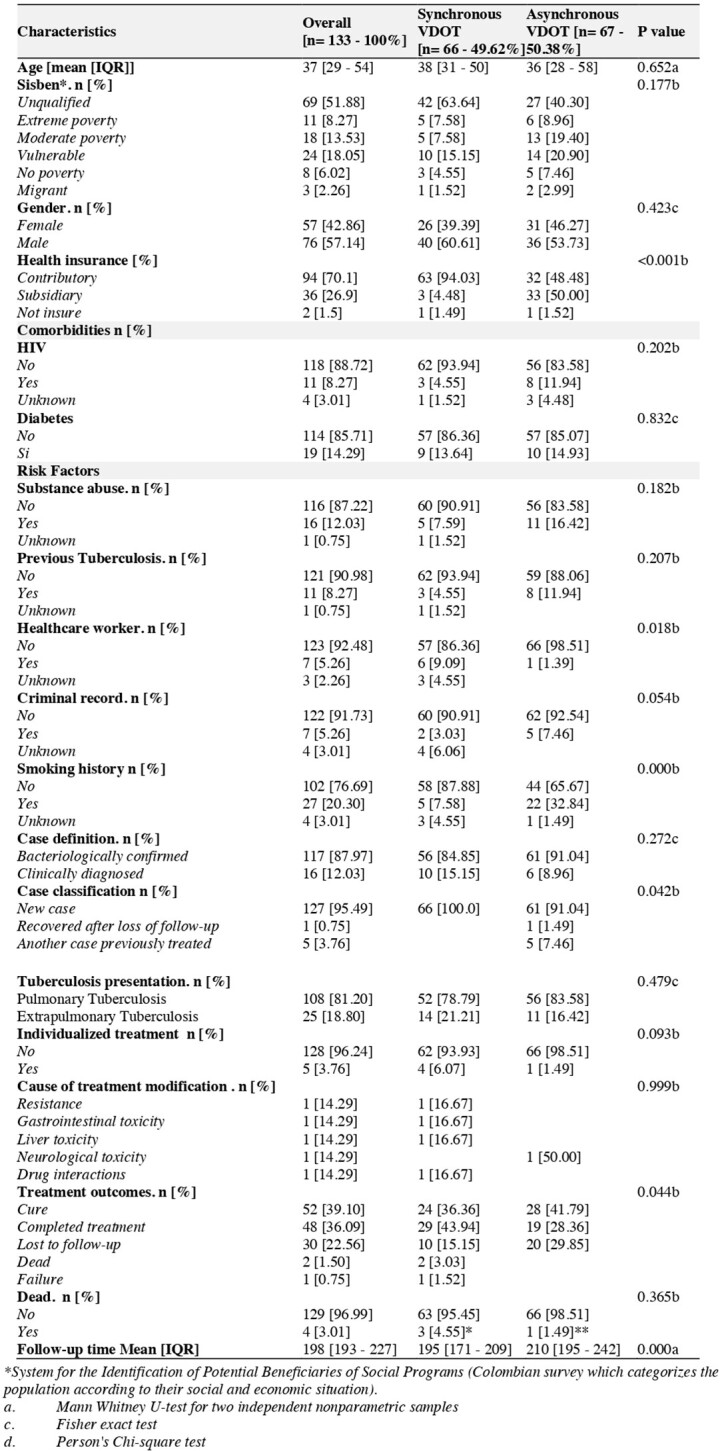
Clinical and demographic characteristics and risk factors

**Methods:**

A quasi-experimental study was conducted in Cali, Colombia. Patients aged ≥ 18 years with TB sensitive to first-line drugs were included. Patients were allocated in synchronous VDOT, where remote and real-time video calls were daily, while in asynchronous VDOT, patients sent video messages of the daily dose intake using the WhatsApp app mobile application. The characteristics of both groups were described, and the comparison was made according to the intervention (VDOT) using Cox regression.

**Results:**

A total of 133 were included, 66 in synchronous VDOT and 67 in asynchronous VDOT . The mean age was similar in both groups and, the 60% of the patients were male. Patients in the asynchronous VDOT group had more comorbidities. Pulmonary tuberculosis was the most frequent presentation in both groups. Treatment was completed in 43.9% in synchronous VDOT, and 28.3% in asynchronous VDOT. Loss to follow-up was higher in the asynchronous VDOT. Four deaths occurred in synchronous VDOT and one in asynchronous VDOT. The mean follow-up time was 198 days. Also, Cox regression showed that the synchronous strategy has 88% risk of completing antituberculosis treatment in less time than the asynchronous monitoring group, HR: 1.88 IC95%: 1.26 - 2.81, p= 0.002.

**Conclusion:**

Asynchronous VDOT seems to have a higher risk of desertion than synchronous VDOT, as opposed to literature finds. A relationship between comorbidities or risk factors that have not been evidenced in our study is not ruled out. Further studies are needed to compare the effectiveness of synchronous vs asynchronous VDOT in challenging scenarios, such as low income, poor internet connectivity, poor technology, and limited logistical capacity of the TB group.

**Disclosures:**

**All Authors**: No reported disclosures

